# Treatment of Tourette syndrome by acupuncture combined with Chinese medicine based on syndrome differentiation: A review

**DOI:** 10.1097/MD.0000000000034268

**Published:** 2023-07-21

**Authors:** Kexin Lin, Yijie Wang, Jiaqi Wang, Chuanyu Zhang, Qiuju Feng

**Affiliations:** a Heilongjiang University of Chinese Medicine, Harbin, China; b The Second Affiliated Hospital of Heilongjiang University of Traditional Chinese Medicine, Harbin, China.

**Keywords:** acupuncture combination of acupuncture and medicine, to twitch, Tourette syndrome, traditional Chinese medicine

## Abstract

Tourette syndrome (TS) is a chronic neurodevelopmental disorder characterized by involuntary motor and speech tics, which can greatly reduce the quality of life of patients. The pathophysiology of TS involves both genetic and environmental factors. Assessing TS pathogenesis is complex, and its underlying pathophysiology is not fully understood. It is gratifying that the research in the past 5 years has brought new research progress on the genetic, neurophysiological and brain network changes of TS. However, despite the progress of research, the treatment methods and drugs of modern medicine are still unsatisfactory, and it is difficult to achieve satisfactory results. Traditional Chinese medicine, as a part of complementary and alternative medicine, has unique efficacy in the treatment of TS, and the safety of its treatment is also worthy of attention. Based on the latest achievements in the pathophysiology of TS, this article will discuss the treatment of TS by acupuncture combined with medicine.

## 1. Introduction

Tourette syndrome (TS) is a childhood-onset neurodevelopmental disorder. TS is a frequently observed neuropsychiatric disorder in children of all ethnic backgrounds,^[1]^ characterized by involuntary movements (motor tics) and vocalizations (vocal tics).^[2]^ TS is estimated to be diagnosed in 0.3% to 0.9% of school-aged children^[3]^ and 0.002% to 0.08% of adults,^[4]^ but there are few adult epidemiological studies worldwide. According to the latest national survey,^[5]^ the latest estimate of TS prevalence in China is 2.46%. TS occurs mostly in men, with a ratio of 4:1, and the symptoms tend to be more severe in men than in women.

Tic and tic disorders are familiar to most pediatricians, neurologists, and psychiatrists, and the underlying neuronal mechanisms should be considered in the treatment of TS. Because the underlying neuronal system of TS involves both environmental and genetic control systems,^[6,7]^ the diagnosis, treatment, and study of its associated comorbidities are complex. The severity of symptoms and comorbidities was highly heterogeneous and fluctuated over time. Although the pathophysiology of TS is not fully understood, various genetic and neurophysiological factors may be responsible. Established treatments for TS include behavioral therapies and medications, while emerging treatments include noninvasive neuromodulation and deep brain stimulation. There are still many gaps in knowledge about TS, including factors that contribute to variability in clinical presentation and how best to treat tics and comorbidities. In this review, the author will summarize the latest evidence on the clinical features, diagnosis, pathophysiology, and treatment of TS with traditional Chinese medicine and acupuncture, and highlight the issues and topics for future research.

## 2. Clinical features of TS

TS is a motor disorder associated with a wide range of psychological and behavioral disorders.^[7,8]^ Among the various motor disorders in children, tics are the most common. Emilio Fernandez-Alvarez and Jean Aicardi reported twitching in about 40% of nearly 700 cases.^[9]^

Tics are divided into motor tics and vocal tics, each of which is divided into simple and complex tics. According to the type of tic and the duration of tic occurrence, TS can be divided into transient tics, chronic motor tics, chronic vocal tics and various chronic tics associated with vocal tics.^[10]^

The clinical features of TS are age-related. TS can be transmitted at any age in childhood, but generally at about 2 to 18 years old, with a peak at about 6 years old.^[11,12]^ Motor tics tend to start earliest, from simple to complex; while vocal tics tend to start later than motor tics, from simple to complex.^[7,13]^ The peak severity of TS tends to be in the second decade. However, TS may persist into the late teens and even into adulthood,^[14]^ and natural processes show ebb and flow in the characteristics of TS type and severity.

Researchers have observed premonitory impulsivity or premonitory sensory phenomena. TS is more common in men and women, and is often associated with comorbidities such as attention deficit hyperactivity disorder (ADHD) and obsessive-compulsive disorder (OCD), in which ADHD has an earlier onset and OCD tends to have a later onset.^[7,13]^ TS is also associated with other psychiatric, behavioral, and neurological disorders, including aggression, self-harm, panic disorder, depression, autism spectrum disorder, restless leg syndrome, and migraine.^[8]^ Age-related occurrence and clinical course are important, and changes in surface pathophysiology are age-related.^[6]^

## 3. Diagnosis of TS

The diagnosis of TS is made through a clinical assessment of the person’s medical history, and the United States Diagnostic and Statistical Manual of Mental Disorders States that for a diagnosis of TS, tics must have started before the age of 18, and the patient must have had tics for at least 1 year, including at least 2 motor tics and 1 speech tic. Diagnostic and Statistical Manual of Mental Disorders-5 distinguishes between transient tic disorders, persistent (chronic) tic disorders, and TS: however, it has been proposed to define these disorders as a spectrum (listed from lightest to heaviest).^[15,16]^ A family history of tics or TS is also helpful in the diagnosis, but it is not a requirement.

Several rating scales are available to assess the symptoms of TS patients in any age group,^[17]^ but the Yale Global Tic Severity Scale (YGTSS) is the most commonly used tool. Given the diversity and volatility of symptoms, European clinical guidelines for TS and other tic disorders-version 2.0^[18]^ suggests several methods to measure the severity of symptoms, Include direct observation in the in-and-out clinic environment, historical information from individuals and families, and video-based assessments.

The diagnosis and evaluation of TS can be challenging due to the clinical heterogeneity of TS, the suppressibility of tics, and the fluctuation of symptoms over time and across conditions. Diagnosis can be delayed up to 3 to 11 years after the onset of symptoms, and an estimated 73% of patients are initially misdiagnosed, but this estimate is based on patient self-report, suggesting that the general community and health professionals have inadequate awareness and knowledge of how to identify TS. However, there is some evidence that people with specific characteristics are likely to develop TS. For example, a multicenter study showed that children with conduct problems, autism spectrum disorder symptoms, OCD, and emotional problems may be more likely to develop tics.^[19]^ Increased awareness of TS and its risk factors can lead caregivers and health care providers to recognize TS and seek early medical attention.

## 4. Time course of TS symptoms

The time course of TS is variable, with tics usually beginning in individuals aged 3 to 8 years, often beginning with simple motor tics followed by vocal tics. The severity of tics typically peaks in individuals aged 8 to 12 years.^[12]^ A single-center study of 1032 individuals^[20]^ reported that the ability to anticipate emotional impulses and inhibit tics can also occur before and after the onset of tics. Tic severity and most associated psychiatric disorders improve with age.^[21]^ However, a prospective longitudinal follow-up study of 314 TS patients^[22]^ showed that only 17% of patients would have complete remission after the age of 16 years, and 60% of patients would still have some persistent mild to moderate tics. Up to 23% of patients will have persistent tics, and in some cases severe tics, well into adulthood.

Predictors of the time course of TS have not been well established, and few longitudinal large cohort studies have been conducted on the severity of tics in childhood^[23]^ (but not the age at which tics occur^[20]^), family history of TS, the presence of comorbidities, possible ridicule for symptoms in childhood, and psychosocial stress. May predict tic severity in early adulthood.^[23,24]^ Identifying predictors of who will develop severe symptoms in adulthood is critical to developing an effective treatment plan and ensuring that these individuals receive the support they need in adulthood.

## 5. Complications of TS

TS often co-occurs with other psychiatric and behavioral disorders, including compulsive behaviors or OCD, ADHD, autism spectrum disorders, anxiety disorders, depression, sleep disorders, and self-injurious behaviors. A cross-sectional study of 1374 people showed that isolated TS is the exception rather than the rule: up to 88% of TS patients are diagnosed with at least 1 additional psychiatric disorder in their lifetime, and 58% will be diagnosed with two or more comorbidities.^[25]^ The most common comorbidities were ADHD and OCD, with an estimated 72% of TS patients diagnosed with both.

Although the time varies from person to person, the onset of ADHD may be the earliest, followed by OCD, anxiety disorder and mood disorder.^[25]^ Furthermore, consistent with other psychiatric disorders, patients with TS and comorbidities have an increased risk of death from natural or unnatural causes compared to TS patients without comorbidities, but the contributing factors are unclear.^[26]^ Evaluation of children with TS for comorbid behavioral and psychiatric symptoms should be initiated early and repeated regularly because comorbidities have a greater adverse impact on quality of life than TS.

## 6. Genome-wide association studies and other genetic determinants of TS

Although TS is highly heritable,^[27]^ variants of known TS risk genes such as CNTN6, NRXN1, SLITRK1, HDC, and CELSR3 are present in less than 2% of affected individuals.^[28–31]^ TS is highly polygenic, and it has been confirmed that a variety of common genetic variants with small effects are widely distributed in the genome.^[32]^ Therefore, genome-wide association studies (GWASs)^[33]^ would be beneficial to further elucidate the underlying genetic etiology of the disease.

To date, only 1 GWAS study of TS has been published.^[34]^ Although no single nucleotide polymorphisms (SNPs) met the criteria for genome-wide significance (*P* < 5 × 10^−8^), but in general, the top SNPs (*P* < 1 × 10^−3^) were enriched in the eQTLs in the frontal cortex and the mQTLs in the cerebellum, suggesting that a significant proportion of these variants have biological relevance to TS and may be associated with other TSs. However, as with other neuropsychiatric disorders, a larger sample size is needed to elucidate the genetic basis of the disorder.

A recent GWAS meta-analysis included 8265319 SNPs from 4819 TS case subjects and 9488 control subjects.^[35]^ No evidence of residual population stratification or systematic technical artifacts was observed in any of the individual data sets or the final meta-analysis (λ = 1.072, λ1000 = 1.011). LDSC showed that 86% of the observed test statistics were inflated to be attributable to an underlying genome-wide polygenic signal. The PRS analysis of each individual GWAS dataset, using the skimming method, and the deCODE sample, showed that all datasets were genetically coexistent.

The first SNP in the GWAS meta-analysis, rs2504235 on chromosome 13q12.2, exceeded the genome-wide significance threshold (odds ratio = 1.16, *P* = 2.1 × 10^−8^). No other SNPs reached genome-wide significance, although rs2504235, a common FLT3 missense variant (Thr227Met) located 11.4 kb away, had a strong LD with rs2504235 (*R*^2^ = 0.93) with a *P* value of 8.2 × 10^−8^. Across the genome, 39 LD-irrelevant SNPs with *P* values <1 × 10^−5^ were identified by LD pruning (*R*^2^ < 0.2), followed by conditional association analysis, controlling for the most important SNP within each 2 Mb window, and manual detection of regional association maps to confirm the presence of supportive statistical evidence of association for nearby SNPs.

While genetic and familial factors are likely to influence the development of brain pathways and manifestations of TS, the precise mechanisms of these interactions are unknown. However, whole-exon studies have shown that several cellular processes are involved in TS pathophysiology, including genes regulating cell polarity and migration,^[30,36]^ cell adhesion molecules involved in transsynaptic signaling, ion channel signaling via γ– aminobutyric acid and glutamate neurotransmitters, glial-derived neuroimmunity,^[37]^ and synaptic membrane stability.^[38]^ A genome-wide cellular and tissue enrichment analysis of a database of 714 healthy human donors revealed that regulation of the expression of numerous genes by non-coding variants may be a key mechanism in the pathogenesis of TS, and that the brain regions most affected by TS-related genes are the dorsolateral frontal cortex, followed by the frontal cortex, striatum, and cerebellum.

New progress has been made in neuroimmunity, which is of special significance to TS, because neurodevelopmental disorders may be caused by external factors (such as maternal inflammation leading to fetal neuroinflammation)^[39]^ and the disruption of genetic neuroimmune pathways caused by gene variation. Some peripheral markers, such as type 1 and type 2 cells, are related to the pathogenesis of autoimmune diseases. Alterations in immune-brain crosstalk in TS are also supported, suggesting that immunomodulation may be a promising therapeutic Avenue.

## 7. Pathophysiology of TS

Consistent evidence from genetics and neuroimaging strongly supports that TS is a neurodevelopmental disorder. Neuroimaging studies have shown differences in brain functional connectivity (i.e., a statistically significant measure of brain activity derived from functional imaging at rest) when adults and children with TS are compared.^[40]^ In addition, the developmental trajectories of TS patients appear to be different, with children with TS showing older brain functional connectivity compared with age-matched controls, while adults with TS show less brain functional connectivity. This difference may be due to differences in cell and axon pruning, which may be influenced by environmental and genetic factors.^[41]^ Overall, genetic and environmental factors may contribute to a wide range of neuronal network dysfunction, and symptoms related to TS can occur.

At the network level, tics may be the product of dysfunction of inhibition within the sensorimotor cortex-basal ganglia network.^[42]^ Changes in the inhibitory microcirculation from the striatum and problems with autoinhibitory action. In contrast, volitional inhibition, measured as active inhibition (inhibition of actions in preparation) or reactive inhibition (inhibition as well as initiation of actions), is largely unchanged in TS patients^[43]^ and is related to the individual’s ability to inhibit tics.

However, the inhibition model can not explain some basic characteristics of TS, such as the fluctuation characteristics of twitch and the preperception characteristics. Another hypothesis is that tics may represent exaggerated and persistent motor habits that are reinforced by abnormally increased phasic dopamine release.^[44]^ In support of this idea, dopamine-related reward-guided learning, also known as reinforcement learning, appears to be enhanced in TS.^[44]^ Tic, as a habitual and learned action, may also account for preaffective impulses; termination of preaffective impulses by the execution of tics may trigger positive prediction errors and episodic dopamine release that reinforce tic learning. The current presensory impulse model proposes that aberrant intrinsic and extrinsic sensory processing may generate presensory impulses that lead to the initiation of behavior, followed by the execution of tics through the cortico-basal ganglia sensorimotor network.

All of these studies suggest that TS patients have increased low-frequency power (1–10 Hz) activity in the central thalamus, which may be related to tics. Other studies have also shown low-frequency activity in the anterior globus pallidus of TS patients.^[45–48]^ How this low-frequency activity is converted into tics is unknown, but low-frequency power in the anterior globus pallidus may be related to presensory impulses.^[49]^

Other hypotheses related to the pathogenesis of TS include that TS is a disorder of social behavioral networks, and that face perception in patients with TS causes abnormally high activity in insular cortex and in neural networks related to tic production, including motor cortex regions and putamen, compared to healthy controls.^[50]^ Another important clue was the study of sensory-action bonding, which showed that the strength of bonding between sensation and action was higher in TS patients than in healthy controls, which also correlated with the severity of tics. Analysis of electroencephalogram signals in an event file encoding task to measure the combination of perception and action showed that this effect was related to activity in the inferior parietal cortex, suggesting that tics may be an evoked response to internal or even external stimuli.

## 8. TCM cognition of TS

There is no corresponding disease name of TS in ancient books of traditional Chinese medicine, and there is also a lack of systematic description. However, the symptoms of repeated involuntary tics in children associated with TS, such as nodding, blinking, mouth opening, shoulder lifting, leg lifting and belly bulging, have been similarly described in some ancient literature.

In traditional Chinese medicine, this disease is classified into “spasm syndrome” and “liver wind” syndrome differentiation and treatment. As early as in the era of Neijing, there are many descriptions about spasm syndrome, such as Su Wen Zhi Zhen Yao Da Lun, which States that “all spasms are strong and belong to dampness.” It is also mentioned in the same article that “all violence and violence belong to the wind.” It is said in Plain Questions on the Generation of the Five Zang Organs: “When a person lies down, blood returns to the liver. The liver receives blood and can see, the feet receive blood and can walk, the palms receive blood and can grasp, and the fingers receive blood and are able to absorb.”

In the Eastern Han Dynasty, Zhang Zhongjing first discussed spasm syndrome in his book Synopsis of the Golden Chamber. In the Ming and Qing Dynasties, the understanding of spasm syndrome in traditional Chinese medicine made rapid progress, and many new theories and viewpoints were put forward. Ye Tianshi in the Qing Dynasty believed that spasm syndrome was closely related to the liver, and that the liver was a rigid viscera, governing the tendons, and its nature was ascending and active.

In the theory of traditional Chinese medicine, there are still different understandings of TS. Professor Huang Linna^[51]^ recorded according to Tremor in Compendium of Medicine: “Tremor is shaking; vibration is moving..” The multiplication of wind and fire, the image of shaking, combined with the clinical manifestations of this disease, it belongs to the category of “tremor syndrome.” Professor Li believes that according to its clinical manifestations, it is similar to the wind syndrome in traditional Chinese medicine, which belongs to the category of “convulsion,” “twitch,” “muscle” and other diseases.^[52]^

## 9. Etiology and pathogenesis of TS

At present, there is no unified understanding of the etiology and pathogenesis of TS in the field of traditional Chinese medicine, and there is no consensus on diagnosis and treatment. Based on the current clinical knowledge,^[51,53]^ Professor Zhuang and Professor Huang summarized the etiology and pathogenesis of TS as follows:

The location of TS is mainly in the liver, which is closely related to the heart, spleen and kidney.^[53]^ The etiology of TS is multifaceted, which is related to congenital deficiency, birth injury, asphyxia, feeling of exogenous pathogens, emotional disorders, etc. It is mostly caused by excessive 5 emotions and internal disturbance of wind-phlegm. The specific pathogenesis is as follows.

### 9.1. Qi depression transforming into fire

“People have five internal organs, which are transformed into five qi, and lead to joy, anger, sorrow and fear.” The liver is in charge of smoothing the flow of qi, and its nature is smooth. Qi depression transforms into fire, which consumes yin essence. If the liver blood is insufficient, the tendons and vessels will lose nourishment, and the deficiency wind will move internally, so the head will stretch and the brain will shrink, and the limbs will tremble.

### 9.2. Accumulation of phlegm due to spleen deficiency

Deficiency of natural endowment or malnutrition after illness, damage to the spleen and stomach, failure of the spleen to transport, retention of water, accumulation of phlegm, accumulation of phlegm and qi, obstruction of the chest, and deficiency of both the heart and spleen are the syndromes.

### 9.3. Liver-yang transforming into wind

“Yang is often excessive, Yin is often insufficient,” which is the physiological basis of this disease in children. There is a saying in Pediatric Medicine Zheng Zhi Jue: The Liver Has Wind: “Every disease, whether new or long, leads to liver wind, and the wind moves and stops at the head..” Professor Huang agrees that the core of this disease should be the liver, which is the rigid viscera, and children are “the body of pure Yang.” If there is deficiency of essence and blood, it is easy to cause liver wind, just as it is said in Su Wen · Yin Yang Ying Xiang Da Lun that “when wind prevails, it will move,” which can cause various twitching symptoms.^[54]^

### 9.4. Wind due to yin deficiency

The liver uses Yang instead of yin, governs the flow of qi and stores blood, and has the function of dredging qi, blood and body fluid of the whole body. Children’s improper diet and overeating of raw, cold, fatty and greasy food will hinder the stomach and cause weakness of the spleen and stomach, resulting in abnormal biochemical restriction of the 5 internal organs and wind due to deficiency.^[55]^ Or due to congenital deficiency, loss of true yin, water does not contain wood, liver Yang is hyperactive, and the hair is twitching.

## 10. Traditional Chinese medicine treatment of TS

Nowadays, there is no unified standard for the syndrome differentiation and analysis of this disease in the field of traditional Chinese medicine, and doctors have different treatment based on syndrome differentiation. Professor Zhuang divided the disease into 6 syndrome types, including hyperactivity of liver-fire, internal movement of liver-wind, invasion of the lung by pathogenic wind, movement of phlegm-heat and hyperactivity of Yang due to spleen deficiency.^[56]^ Professor Li divided the disease into 6 syndrome types, namely, hyperactivity of liver-wind type, phlegm-fire disturbing the spirit type, spleen deficiency and hyperactive liver type, yin deficiency and wind type, latent fire in the spleen and stomach type and wind-phlegm disturbance type.^[52]^ Liu believes that this disease belongs to the syndrome of liver wind and spasm, and the treatment should tonify the congenital, nourish the acquired, and at the same time calm the liver and subdue Yang.^[57]^

### 10.1. TCM treatment practice of TS

Professor Li believes that TS has different types of pathogenic excess and healthy qi deficiency and pathogenic excess,^[52]^ which is marked by wind-fire and phlegm-qi, and its origin is in the liver, spleen and kidney, especially closely related to the liver meridian, so he puts forward “one prescription and six types” on this basis. “Yifang” refers to Pinggan Xifeng Powder: Rhizoma Gastrodiae, Ramulus Uncariae Cum Uncis, Scorpio, Rhizoma Typhonii, Semen Ziziphi Spinosae, Rhizoma Acori Graminei, Dens Draconis (decocted first), Radix Saposhnikoviae, Arisaema Cum Bile, Radix Paeoniae Alba, and Fructus Chaenomelis. The “six types” are as follows: the prescription for hyperactivity of liver and wind is Pinggan Xifeng Powder combined with Xieqing Pill; the prescription for phlegm and fire disturbing the mind is Pinggan Xifeng Powder combined with Mengshi Guntan Pill; the prescription for spleen deficiency and liver hyperactivity is Pingganxifeng Power combined with Gouteng Yigong Powder; the prescription of yin deficiency and wind is Pingganxifeng Power combined with Sanjia Fumai Decoction; the prescriptions for spleen and stomach latent fire are Pinggan; Pinggan Xifeng Powder combined with Yuzhen Powder was selected as the prescription for wind-phlegm disturbance type.

Zheng et al^[58]^ designed a randomized controlled trial. To explore the effect of Wuling Powder (Radix Paeoniae Alba, Rhizoma Gastrodiae, Fructus Tribuli, Ramulus Uncariae cum, Lucid Ganoderma, Caulis Polygoni Multiflori, Semen Ziziphi Spinosae, Fructus Schisandrae Chinensis, Fructus Gardeniae, Efficacy and safety of Rhizoma Arisaematis and Radix Scutellariae in the treatment of TS. The trial randomized 603 TS patients aged 5 to 18 years to treatment with placebo (n = 117), tiapride (n = 123, 400 mg/d), and Wulingsan (5-LGr) (n = 363, 15–22.5 g/d) for 8 weeks. The primary outcome was to compare the incidence of adverse events between the 3 groups using the YGTSS and its subscales, the total tic score, and tic related impairment. Conclusions 5-LGr is clinically as effective as tiapride in reducing convulsions. Its safety is superior to that of Tiapride. 5-LGr can be considered as a safe and effective treatment for TS.

Fan et al^[59]^ designed a randomized controlled trial to explore the clinical efficacy and safety of JXZDF in the treatment of TS. JXZDF is composed of the following drugs: Zhenzhumu (Concha Margaritifera), calcined Longgu (Os Draconis), Muli (Concha Ostreae), Suan zao ren (Semen Ziziphi Spinosae), Baiziren (Semen Platycladi), Baishao (Radix Paeoniae Alba), Jiangcan (Bombyx Batryticatus), Chaihu (Radix Bupleuri), Niubangzi (Fructus Arctii), Zhiqiao (Fructus Aurantii), Dilong (Lumbricus), Chantui (Periostracum Cicadae), and Baizhi (Angelica Dahurica). In this randomized controlled trial, 120 TS patients aged 6-16 years were randomly assigned to receive JXZDF (n = 60, 17.6 g/d) or aripiprazole (n = 60, 10 mg/d) for 12 weeks. The primary outcome was measured using the YGTSS, and adverse events were assessed with the Treatment Emergent Symptom Scale. Conclusion JXZDF is safer than aripiprazole, and its clinical efficacy is not inferior to that of aripiprazole. Another network pharmacology experiment verified^[60]^ that JXZDF can change the activation of microglia and the expression of pro-inflammatory mediators by inactivating PI3K/AKT signaling pathway, which may be one of the mechanisms of JXZDF in the treatment of TS.

Yang designed a randomized controlled trial^[61]^ to observe the clinical effect of Jianpi Zhichou Granule combined with Tiapride in the treatment of TS. He collected TS children who were treated in the Department of Internal Medicine of Children’s Hospital Affiliated to Capital Institute of Pediatrics from March to November 2015, and randomly divided them into control group and treatment group, with 30 cases in each group. The control group was treated with Tiapride alone, while the treatment group was treated with Tiapride combined with Jiannao Zhiquan Granules for 12 weeks. The daily dose of tiapride was recorded, and the YGTSS scores and effective rates were compared between the 2 groups before and after treatment. Conclusion Compared with Tiapride treatment alone, Jiannao Zhichou Granules combined with Tiapride did not significantly improve the effective rate of TS treatment, but could reduce the daily dose of Tiapride and significantly reduce the YGTSS score.

Feng et al^[62,63]^ designed 2 groups of animal experiments to explore the effects of Changpu Yujin Decoction on the synaptic ultrastructure, synaptophysin (SYN) and postsynaptic density 95 (PSD-95) protein of TS rats, and to explore the effects of CPYJD on the synaptosomal SNARE protein complex of TS model rats. Feng Peng: First of all, 240 3-week-old SD rats were selected and divided into blank group (n = 60) and model group (n = 180) according to the random number table. TS animal model was established by intraperitoneal injection of 3,3 ‘-iminodipropionitrile. After successful modeling, the rats were randomly divided into model group, Tiapride group and Changpu Yujin Decoction group, with 60 rats in each group. From the next day after the completion of modeling, the blank group and the model group were given normal saline, and the other groups were given corresponding drugs, once a day, for 4 weeks. At 0, 7, 14, 21, and 28 days, 12 rats were randomly selected from each group. After anesthesia, the striatum was taken out. The synaptosomes in the striatum were prepared by Percoll density gradient centrifugation and identified morphologically under transmission electron microscope. The expression of SNAP-25, Syntaxin-1a and VAMP-2 mRNA and protein in synaptosomes was detected by qPCR and Western blot, and the expression of the above indexes in striatum was detected by immunohistochemistry. Conclusion Changpu Yujin Decoction can promote the release of dopamine by regulating the expression of SNAP-25, Syntaxin-1a and VAMP-2, thus exerting its anti-tic effect. 2. Feng Peng divided 140 SD rats into blank group (35 rats) and model group (105 rats) according to random number table. TS animal model was established by intraperitoneal injection of iminodipropionitrile, and then the model group was randomly divided into model group, tiapride group and CPYJD group, with 35 rats in each group. From the next day after the completion of modeling, the blank group and the model group were given normal saline, and the other groups were given corresponding drugs, once a day, for 4 weeks. Seven rats were randomly selected from each group before treatment and on the 7th, 14th, 21st, and 28th day of treatment, respectively. After behavioral assessment, the striatum was stripped under anesthesia. The synaptic ultrastructure was observed by transmission electron microscope, and the changes of SYN and PSD-95 protein levels in the striatum were detected by immunohistochemistry. Conclusion CPYJD can improve the stereotypy and locomotor behavior of TS rats. Compared with tiapride, CPYJD has a late onset of action, but the anti-tic effect is stable after the onset of action, and its mechanism may be related to the improvement of prominent structure and function, and the increase of SYN and PSD-95 protein expression.

Meta-analysis was designed by Chen et al^[64]^ to systematically evaluate the clinical value of integrated traditional Chinese and western medicine in the treatment of TS. Chen comprehensively searched China HowNet Full-text Database, VIP Chinese Science and Technology Journal Full-text Database, Wanfang Medical Journal Database, China Biomedical Literature Service System and other databases to retrieve randomized controlled trials of traditional Chinese and Western medicine in the treatment of TS, and set the search period from January 1, 2010 to January 1, 2020. According to the modified Jadad scoring criteria, the quality of the final eligible literature was evaluated, and the data of the main outcome indicators in the literature were entered for Meta-analysis by Review Manager 5. 0 software. Conclusion Compared with single western medicine treatment, integrated traditional Chinese and western medicine treatment of TS has more advantages in improving the total effective rate, improving the condition, reducing tic attacks, and will not increase the incidence of adverse reactions, which is safe and reliable.

A meta-analysis of 47 randomized controlled trials^[65]^ showed that Changma Xifeng tablets and Jiuwei Xifeng granules had obvious clinical efficacy in the treatment of TS, but their specific therapeutic effects need more scientific verification.

### 10.2. Practice of acupuncture treatment of TS

Professor Sun believes that TS is a psychosomatic disease,^[66]^ the core of pathogenesis is wind due to blood deficiency, and the location of the disease is in the heart and liver. Therefore, the selection of acupoints should be guided by the functional location of the cerebral cortex, the extrapyramidal area should be the first choice for tic disorders, and the emotional area should be the first choice for behavioral disorders. The treatment focuses on multiple methods to regulate the mind, including manipulation to regulate the mind, using repeated transcranial acupuncture; multiple points to regulate the spirit, taking Baihui, Neiguan, Shenmen and Dazhong. According to the abdominal twitch and mental symptoms of children with TS, the first and third abdominal areas were selected for acupuncture. Local acupoint selection, head and tail acupoint selection, and acupoint selection of the same meridian were used to strengthen the treatment of “symptoms” (target symptoms). Chaihu plus Longgu Muli Decoction and Siwu Decoction were used to soothe the liver, nourish the blood and stop the wind. According to the characteristics of TS and target symptoms, filiform needle, acupuncture, traditional Chinese medicine, psychotherapy and other methods were selected for sequential treatment.

Zhang et al^[67]^ designed the clinical observation, and used the method of regulating the mind (acupuncture at Baihui, Yintang, Shangxing, Zhongwan, Taiyi, Huaroumen, Shenmen, Daling, Shenmai and other acupoints, once every other day). Collected 60 cases of TS (liver wind syndrome) in the outpatient department of Nangang Branch of Heilongjiang Academy of Traditional Chinese Medicine from January to December 2019, and randomly divided them into observation group and control group with 30 cases in each group. The control group was treated with Tiapride Hydrochloride, and the observation group was treated with the method of regulating mental activity combined with acupuncture and medicine, with a course of 8 weeks for both groups. The TCM symptoms and the overall clinical efficacy were observed before and after treatment in the 2 groups, and the YGTSS scores were compared between the 2 groups before and after treatment. Conclusion The method of regulating mental activity combined with acupuncture and medicine can significantly improve the curative effect of TS, improve clinical symptoms and reduce the YGTSS score.

Zhang designed a randomized controlled trial^[68]^ to observe the clinical efficacy of acupuncture combined with haloperidol in the treatment of children with Tourette’s syndrome. Methods 70 children with TS in our hospital from September 2015 to September 2017 were randomly divided into control group and treatment group, 35 cases in each group. The control group was treated with oral haloperidol, while the treatment group was treated with acupuncture (acupuncture at Baihui, Sishencong, Yintang, Shenting, Shuigou, Zusanli, Sanyinjiao, Taichong, Neiguan and Shenmen) on the basis of the treatment method of the control group. After 8 weeks, the clinical efficacy of the 2 groups was statistically analyzed. Conclusion Acupuncture combined with haloperidol in the treatment of children with Gilles de la Tourette’s syndrome has a significant clinical effect, which can effectively improve the YGTSS scores of motor tics and vocal tics, as well as the levels of DA and 5-HT, and is worthy of clinical application.

Sun and Chen^[69]^ designed clinical observation, comparing cluster needling of Yu’s scalp acupoints (anterior parietal area, parietal area and frontal area^[70]^). After routine disinfection of each acupoint, a 0.40 mm × 0.40 mm disposable filiform needle was inserted obliquely into the lower layer of galea aponeurotica from each acupoint of the scalp, and then the needle was inserted in parallel for about 20 to 25 mm. After the arrival of qi, it was quickly twirled for 2 to 3 mm. The frequency is about 200 times/min, and the needle is retained for 6–8 hours.) And haloperidol in the treatment of Tourette’s syndrome. Methods 60 patients were randomly divided into 2 groups, 30 cases in each group. The treatment group was treated with cluster needling of Yu’s scalp points, and the control group was treated with oral haloperidol. The clinical efficacy and the score of YGTSS were observed before and after treatment. Conclusion Cluster needling of Yu’s scalp points has a good therapeutic effect on Gilles de la Tourette’s syndrome, which is safe and effective.

### 10.3. Treatment of TS with acupuncture and medication

In the long-term medical practice, Chinese medicine practitioners have gradually found that although both Chinese medicine prescriptions and acupuncture can produce therapeutic effects on TS, the effect of Chinese medicine combined with acupuncture on TS is more significant, and the adverse reactions are less.^[66]^

Zheng et al^[71]^ designed clinical observation to explore the effect of external treatment combined with Jianpi Pinggan Decoction and Radix Puerariae on immune function and helper T cells (Th) in children with ADHD and TS. Methods One hundred and thirty-five children with ADHD and TS were randomly divided into western medicine group (n = 45, treated with routine western medicine), traditional Chinese medicine group (n = 45, treated with external treatment of Jianpi Pinggan Decoction and Pueraria root), and integrated traditional Chinese and western medicine group in which 45 children were treated with both routine western medicine and external treatment of Jianpi Pinggan Decoction and Pueraria root) for 2 weeks. The levels of prolactin, immune function and Th cytokines were compared among the 3 groups before and after treatment, and the adverse reactions were observed. Adde Radix Puerariae into Jianpi Pinggan decoction: Poria, Rhizoma Dioscoreae, Os Draconis, Fructus Ligustri Lucidi, Fructus Tribuli, Radix Ophiopogonis, Radix Pseudostellariae, Radix Paeoniae alba, Ramulus Uncariae cum Uncis, Rhizoma Acori Graminei, Radix Bupleuri, Rhizoma Atractylodis Macrocephalae, and Radix Puerariae. Acupuncture at Dazhui, Shendao, Taichong, Zusanli and Baihui was selected for external treatment. Conclusion Gegen plus Jianpi Pinggan Decoction combined with external treatment can significantly reduce the serum levels of prolactin level of children with ADHD and TS, improve the immune function of children, and effectively regulate the level of Th cells, with high safety.

Fan et al^[72]^ designed clinical observation to observe the therapeutic effect of acupuncture (Baihui, Sishencong, Dance Tremor Control Zone, Shenting, Dazhui, Bilateral Fengchi, Bilateral Taichong, Bilateral Hegu) combined with Jingshuaikang capsule on TS. The team collected 38 cases and divided them into 2 groups. 19 cases in the treatment group were treated with acupuncture and Jingshuaikang capsule, while 19 cases in the control group were treated with acupuncture only. The curative effects of the 2 groups were compared. Conclusion Acupuncture combined with oral administration of Jingshuaikang capsule is more effective in the treatment of TS.

Kong et al^[73]^ designed clinical observation to observe the clinical efficacy of scalp acupuncture combined with medication in the treatment of TS. Sixty TS patients were randomly divided into scalp acupuncture group (group A), drug group (group B) and scalp acupuncture combined with drug group (group C), with 20 cases in each group. According to the International Standardized Program for Scalp Acupuncture Point Names, group A was treated with acupuncture at the chorea tremor control area, parietotemporal anterior oblique line, temporal anterior line, parietal midline and parietal lateral line 1, and group B was treated with haloperidol. The patients in group C were treated with haloperidol on the basis of group A for 8 consecutive weeks, and the YGTSS was used to compare the symptom scores and curative effects before treatment, 4 weeks after treatment, 8 weeks after treatment, 1 month after follow-up, and 3 months after follow-up. Conclusion Scalp acupuncture combined with medication has a definite therapeutic effect on TS, which is superior to simple scalp acupuncture or simple medication, and provides a basis for clinical treatment of TS.

Jiang et al^[74]^ designed the clinical efficacy observation to observe the acupuncture of head 3 spirits (Shenting, bilateral Benshen, Sishencong, Baihui, bilateral Fengchi) combined with Zhuang medicine point line (operation method: the experimenter holds the Zhuang medicine line with thumb and forefinger, exposing the thread head about 1 cm, igniting the thread head on the alcohol lamp, aiming the thread head of cylindrical spark at the selected acupoint for rapid moxibustion, and the fire is extinguished. Each time of moxibustion is 1 strong, then burn and moxibustion again, each point is 1–3 strong.) Clinical efficacy in the treatment of TS. The team randomly divided 62 TS patients into an observer group and a control group, with 31 patients in each group. The observer group was treated with acupuncture of Tousanshen combined with moxibustion of Zhuang medicine thread, while the control group was treated with tiapride hydrochloride orally, with 14 days as a course of treatment and 4 courses of treatment. YGTSS was used to evaluate the disease condition before and after treatment, and the curative effects were observed. Conclusion Acupuncture at Tousanshen combined with medicated thread moxibustion of Zhuang medicine has a good therapeutic effect on TS and can effectively improve the clinical symptoms of the patients.

## 11. Discussion

This study is based on the pathophysiological progress that has been proved by modern medical research, and the research of traditional Chinese and Western medicine with higher evidence level is selected for analysis. In the above studies, there were no obvious adverse reactions in the process of taking traditional Chinese medicine preparations and acupuncture treatment of TS. However, clinical medical staff should pay close attention to the medication safety of patients and fully understand the medication history and allergy history of patients. Modern medicine for the pathogenesis of TS is not clear, while the treatment is relatively limited, and the efficacy is not satisfactory. As a part of complementary and alternative medicine, traditional Chinese medicine can effectively supplement the deficiencies of modern medical treatment methods. Traditional Chinese medicine syndrome differentiation and treatment of TS has the characteristics of exact curative effect and high safety, which is worthy of clinical promotion. However, at present, there is a lack of high-quality and high-level randomized controlled trials in the field of traditional Chinese medicine treatment of TS in China and even in the world. The author sincerely hopes that more scholars will participate in this research in the future, so as to provide higher-level and higher-quality research data for clinical practice. In addition, after the elimination of tic symptoms, we should arrange the diet and living of patients scientifically and reasonably, avoid excessive excitement, tension and fatigue, and pay attention to the prognosis of patients to prevent the recurrence of the disease.

## Author contributions

**Conceptualization:** Qiuju Feng.

**Data curation:** Kexin Lin, Yijie Wang, Jiaqi Wang, Chuanyu Zhang, Qiuju Feng.

**Formal analysis:** Kexin Lin, Qiuju Feng.

**Funding acquisition:** Kexin Lin.

**Investigation:** Kexin Lin.

**Methodology:** Kexin Lin.

**Resources:** Kexin Lin.

**Software:** Kexin Lin.

**Visualization:** Kexin Lin.

**Writing – original draft:** Kexin Lin.
